# The Effectiveness of an Intervention Programme for Reducing Peer Rejection in Early Childhood Education

**DOI:** 10.3390/children10111826

**Published:** 2023-11-19

**Authors:** Paula Molinero-González, Luis J. Martín-Antón, Miguel Á. Carbonero-Martín, Wendy L. Arteaga-Cedeño, José Luis Rodríguez-Sáez

**Affiliations:** Excellence Research Group GR179 Educational Psychology, Department of Psychology, University of Valladolid, 47011 Valladolid, Spain; paula.molinero@uva.es (P.M.-G.); miguelangel.carbonero@uva.es (M.Á.C.-M.); wendy.arteaga@uva.es (W.L.A.-C.); joseluis.rodriguez@uva.es (J.L.R.-S.)

**Keywords:** peer rejection, early childhood education, early intervention, socioemotional competence, prevention

## Abstract

In the early years of schooling, peer groups are key to fostering students’ overall learning and development. Yet it has been found that around 10% of children suffer from peer rejection in the classroom, with this problem entailing negative consequences both in the short and long term. The problem proves difficult for adults to detect, which usually leads to a delay in measures being taken to intervene and prevent it. This study applies an experimental design with pre-test and post-test measurements in two groups—experimental and control—in order to address the problem of rejection in the early years of schooling. It explores aspects such as sociometric type, degree of victimisation, social and antisocial behaviour, as well as problematic situations among 637 students at six schools. We then implement an intervention programme for socioemotional competence throughout a school year in an effort to improve students’ social skills and relationships, focusing specifically on preventing and reducing the rejection experienced by some of these children. The programme comprises 35 teaching activities and strategies that promote the development of competences for student inclusion (curbing aggression, encouraging teamwork, fostering self-esteem, etc.) and that seek to involve all students, teachers, and relatives by offering an array of complementary resources that enrich the initiatives applied (a programme calendar, assessment notebook, questionnaires, related website, material resources). After the intervention programme, it was found that the experimental group had reduced the percentage of students who suffered rejection from 9.9% to 7.3%, although the same was not true of the control group, which went from 9.5% of rejected students to 10.2%. The reduction in the percentage of rejected students in the experimental group after the application of the programme is an encouraging result that invites us to continue working on more comprehensive interventions to prevent and reduce this phenomenon.

## 1. Introduction

Childhood and adolescence are key stages in learning and practising the social skills [1] and specific behaviours required to successfully carry out interpersonal tasks, such as making friends [2]. These social relationships and interactions are pivotal to the healthy development of students and to their socialisation [3], with the classroom environment being a key element in providing quality education [4] and with peer groups proving vital [5] in terms of offering possibilities and interests that promote different types of learning and strategies that are crucial for children’s first educational experiences in early childhood education [6,7]. A cordial atmosphere in schools thus has a major impact on the type and quality of learning that can be offered [8]. However, not all children have the same possibilities for interacting and learning, since the sociometric distribution within a classroom can vary enormously and may contain children who are popular, rejected, average, neglected, and controversial [9,10]. Statistically, between 10–15% of students in any given classroom suffer peer rejection [11]. This is a major problem since it is a stressful and painful event, which causes a distortion in their social self-perception and affects their emotional state [12] in addition to impacting their relationship with the environment [13]. Several authors identify similar percentages of rejected students in the first years of primary education; 13% of students, according to Monjas et al. [14], or 9.1% [15]. However, we found fewer studies on classroom sociometric typology in early childhood education, in which 8.1% of students are rejected [11]. It has also been found that this phenomenon mainly affects boys [16], with the latter being more likely to be victims of bullying than girls [17], who are generally more preferred in the classroom [15,16]. It is also important to mention that students with specific educational support needs (SEN) are more prone to suffer rejection [14], and that this increases as they progress through the education system [18].

Students with a low level of acceptance in their peer group tend to exhibit fewer socially accepted responses and to display more aggressive responses [19]. This causes several short- and long-term consequences such as [20] socioemotional difficulties (loneliness, isolation, low performance), internalised problems (low self-esteem, anxiety, depression), and externalised problems (behavioural problems or antisocial behaviour). The frustration they experience may lead to them giving up on their learning activities [21]. Indeed, experiencing unfavourable relationships has even been negatively linked to the emotional, social, behavioural, and neural regulation of the child and future adult [20,22].

Identifying a common pattern of rejected students proves complex because, as several authors [23] point out, there is no exact prototypical profile of a rejected child, although most do display one or more of the patterns of behaviour identified [24], which are the following: low prosocial behaviour; highly aggressive or disruptive behaviour; highly immature behaviour and a lack of attention; and high social anxiety and avoidance behaviour. Amongst the youngest children, patterns of highly aggressive or disruptive behaviour, as well as highly immature behaviour and a lack of attention, might all be seen as one and the same, since expressing aggression is shaped by their inability to stifle impulses and to deal with frustration, with aggression emerging as one of the main causes of rejection in childhood [25]. This coincides with the reasons students cite, in the early stages of education, for not choosing certain children as friends [26,27]. This situation may also be caused by excessive disruptive behaviour [28] or even by a deficit in language skills [29]. Rejected children tend to be less prosocial and to display a more negative self-evaluation [10,30], with students who exhibit low self-esteem being those who are at greatest risk of social exclusion [31], as this is a very influential construct in children’s social development [32].

Although we are aware that not all the conflicts found in schools are equally important or prevalent [33], peer rejection is one of the main forms of ill-treatment to have been identified [34]. Nevertheless, we encountered several problems: the first is that, unlike school bullying, it is not a visible problem and teachers are not always aware of the reality experienced by their students [35]. Furthermore, the younger the students, the more complex it is to identify their situation in the classroom [36], since the infant stage requires investing much time and adaptations to investigate and obtain information from children. As mentioned, research into how students perceive the acceptance and rejection they receive from their peers is scarce, despite such perception being a very valuable social skill vis à vis everyday social interactions [37]. One of the most widely used techniques when evaluating the preference for peers as playmates or friends is peer nomination [26,38,39], which involves accessing the social network structure in the classroom at a given moment [40], for which photographs of students in the early stages of education are very useful for elaborating sociograms.

Several rejected students may express a negative opinion of their schools as a result of the situations they suffer due to their sociometric status [18]. These students are sometimes aware of acts of aggression committed by one peer towards another at school [41], which is defined as the degree of perceived victimisation. This victimisation may be manifested directly—through aggression (verbal and physical)—and indirectly—through more subtle forms such as social exclusion from the group [42]. Students may even suffer problems of depression more frequently than is the case amongst non-victimised children [43]. The children who are most victimised tend to experience greater social rejection and isolation from their classmates [44], with special needs students feeling even more victimised [14]. It is therefore important to assess whether students in the early stages of education are able to identify the degree of their victimisation; i.e., whether they perceive direct or indirect acts of aggression from their peers.

Taking into account the situation regarding rejection, it is vital to intervene from an early age, when the problem of rejection is not yet critical—since social groups are still changing [13]—in order to encourage within students a level of social competence that can prevent antisocial behaviour and foster the right atmosphere for coexistence [45]. Research in this regard shows us that it is necessary to boost children’s relationships and to foster an educational perspective towards friendship [46]. Some studies indicate that it is essential for teachers to take into account different group strategies [47] or for the teacher to know their students’ preferences in order to propose shared games and dynamics that ensure the participation of the whole class [48].

It is essential to implement programmes that help control aggressive and annoying behaviours, thereby deepening self-knowledge and emotional self-control [49] and promoting prosocial skills as these help children to become more popular [50]. These prosocial behaviours should be promoted from childhood, through working on social skills with specific programmes such as those implemented by Monjas [51,52]. Other studies indicate that families are a fundamental axis, because their involvement is an important part of emotional education as a protective factor in conflict situations [53]. It is crucial to remember that a child’s emotional state in early childhood education is directly linked to their behaviour [54].

Scientific research has shown that implementing programmes to prevent bullying can prove very positive [55]. In order to increase research that favours the psychosocial development of rejected students [56]—and taking into account the main characteristics of the aforementioned interventions—this study seeks to gain an insight into sociometric types and to identify which profiles suffer rejection—or which might suffer it—in order to implement an intervention programme about social-emotional competence designed to prevent and reduce the problem. This aim is pursued through the following specific aims:

- To examine students’ sociometric distribution: popular, average, rejected, neglected, and controversial.
- To explore the links between rejection and other personal and contextual variables (victimisation, social behaviour, antisocial behaviour, etc.), providing information to teachers about their students and classes.
- To provide teaching staff with a comprehensively designed socioemotional intervention programme and to apply it as a resource to prevent and reduce rejection in the classroom.
- To compare the results in the experimental and control groups to evaluate the effectiveness of the programme.

## 2. Materials and Methods

### 2.1. Participants

The sample is made up of 637 children in the second cycle of early childhood education (3–6 years old) in six schools, across 33 classrooms. Four of the schools are public and two are semi-private (private schools that receive public funding). In total, 31.7% of the children are in their first year of infant school, 31.1% are in their second year of infant school, and 37.2% in their third year of infant school. A total of 34.7% of the children are new to the school (as a result of being in the first year of early childhood education or because they have recently joined), while 65.3% have already been at school for at least one year. Three schools make up the experimental group—accounting for 60.3% of the students—while the remaining 39.7% are the three schools in the control group (Table 1). With respect to the equivalence between the experimental group and the control group, there are no statistically significant differences in terms of gender distribution, χ^2^ (1, *N* = 637) = 1.499, *p* = 0.225, school year, χ^2^ (2, *N* = 637) = 0.119, *p* = 0.942, or whether or not the students had specific educational support needs (SEN), χ^2^ (1, *N* = 637) = 1.385, *p* = 0.629.

With regard to gender distribution, 52.7% of the students are boys and 47.3% are girls, with 6.8% of the students having some type of special educational needs. Finally, the study involved the participation of 33 teachers.

### 2.2. Instruments

1. Sociometric Questionnaire on Peer Nominations. GREI, 2009 [57]. This is a peer nomination tool which seeks to identify each student’s sociometric type: popular, rejected, average, neglected, and controversial. To achieve this, students must choose which classmates they would like to be with and those they would not. There is no limit on the number of nominations in the group. Given the participants’ age, the questionnaire was adapted to a question–answer game in the form of an individual interview in which students are shown a picture of their personalised school bus. When shown the photographs of their friends and classmates, each student is told that they can take with them those pupils that they would like to go on a trip with, and that they can remove those they do not like, giving the reasons for their choice. This was applied in the pre-test and post-test stages.
2. Victimisation Scale. GREI, 2014 [58]: an eight-item self-report questionnaire in which each child is asked to state how often, from 1 (never) to 5 (almost every day), over the last month they have experienced situations involving possible bullying and victimisation from classmates. The original scale presents a single-factor structure, with suitable psychometric properties: S-B χ^2^ (20) = 26.23, *p* = 0.158; S-B χ^2^ /df = 1.31, CFI = 0.99, BBNN = 0.98, RMSEA = 0.038, 90% CI [0.000, 0.068], and acceptable reliability (alpha = 0.82). In order to apply it to early childhood education, the scale was adapted to a story format with questions. At this educational stage, it also presents suitable psychometric properties, S-B χ^2^ (13) = 19.08, *p* = 0.125; S-B χ^2^ /df = 1.47, CFI = 0.99, NNFI = 0.98, RMSEA = 0.030, 90% CI [0.015, 0.040], although with a two-factor structure that accounts for 74% of variance: (a) direct victimisation, e.g., some classmates treat you badly or make you cry, with an Alpha reliability = 0.93; (b) indirect victimisation, e.g., some classmates do not let you take part in the games and do not want to be with you (Alpha = 0.89). This was applied in the pre-test and post-test stages.
3. Preschool and Kindergarten Behaviour Scale—PKBS-2 ([59], adapted by [60]). This instrument is filled in by the teachers; one for each child. It contains 34 items that measure social behaviour and 44 items that measure antisocial behaviour on a four-point Likert-type response scale, ranging from 0 (never) to 3 (often). The instrument presents a suitable fit, and also displays suitable reliability indices. It was applied to all the students in the pre-test stage, and in the post-test stage it was applied to those students who were sociometrically rejected.
4. Taxonomy of Problematic Social Situations. TOPS ([61], adapted by [13]). This evaluates social situations in which each of the students has problems. The original instrument comprises 44 items on a five-point Likert-type scale, ranging from 1 (never) to 5 (almost always), and it is filled in by the teacher. Items are grouped into six factors, with a high degree of internal consistency (α total = 0.98): (a) inclusion in the peer group (five items, α = 0.95), (b) response to failure (nine items, α = 0.95), (c) response to success (three items, α = 0.89), social expectations (11 items, α = 0.94), and (d) teacher’s expectations (six items, α = 0.95). A reduced version was applied—adapted to younger students [13]—which measures problematic situations via four factors: (a) being disadvantaged, (b) respect for authority and the rules, (c) response to one’s own success, and (d) tworosocial and empathic behaviour. The indices show an excellent fit: χ^2^ (113) = 132.41, *p* < 0.101; S-B χ^2^ /df = 1.17, CFI = 0.99, NFI = 0.97, NNFI = 0.99, RMSEA = 0.032, 90% CI [0.000, 0.052]. The fit and reliability indices—measured with the standardised Cronbach Alpha coefficient = 0.92—are very high. It was applied to all the students in the pre-test stage, and in the post-test stage it was applied only to those students who were rejected.
5. Individual identification sheet: this is an ad hoc instrument that contains information concerning the needs of each child, how regularly they attend school, as well as other open questions that teaching staff can answer in order to gain a deeper knowledge of their students (social relations in the classroom, which students they are most concerned about, overall classroom behaviour, etc.). This was completed in the form of a researcher–teacher interview.
6. Programme evaluation questionnaire: this ad hoc instrument contains four open questions, with 17 specific items on a four-point Likert-type scale, ranging from 1 (do not agree at all) to 4 (totally agree), as well as a general evaluation, ranging from 1 (not at all satisfied) to 5 (totally satisfied). The questionnaire is an evaluation resource, created ad hoc, to analyse teacher satisfaction with the programme at the end of the study. The design of the questionnaire was subjected to the judgement of three experts in order to improve its reliability and validity. It was carried out using Microsoft Forms.

### 2.3. Procedure and Data Analysis

The research was adapted to an experimental design with pre-test and post-test measurements. After a letter had been, sent detailing the study, centres were selected randomly from those that responded positively to the invitation to participate. The study was approved by a Research Ethics Committee (CEIM, code 21-2335 NO HCUV). Authorisation was also received from the Regional Directorate General for Innovation and Teacher Training (Regional Ministry of Education at the Regional Government of Castilla y Leon), as well as from the schools themselves. When necessary, permission for the applicable COVID protocol was also obtained, as was the authorisation and informed consent of all the students’ families. Authorisation to participate in the study was granted for 90.3% of the students.

Two informative sessions were arranged for the teachers and school management staff. The first was aimed at conceptualising peer rejection, and key information was provided concerning the issue and its related variables. The second session looked at the proposed study, detailing its commitments to the school and explaining the instruments, activities, and proposed strategies. The intervention programme also sets out a third voluntary meeting that was held with the families on six occasions.

The study was carried out in three stages throughout one school year.

#### 2.3.1. Initial Session (Pre-Test)

Pre-test data were collected through individual interviews during lesson time in a room near the classroom, in an open environment with no distractions and following the order of the children in the class.

#### 2.3.2. Application of the Intervention Programme for Socioemotional Competence and Teaching Strategies

The intervention programme consists of an array of activities that merge proposals and experiences from different sources concerning referents in peer rejection [51,52,62] together with other innovative proposals, taking into account prior studies on the issue and the application of a previous pilot programme [63], aimed at enhancing its design and implementation.

- Number of sessions: 35.
- Content: curbing aggression and encouraging calm, developing social skills, removing prejudices, improving communication and self-esteem, boosting teamwork, etc.
- Duration: eight months (October–May).
- Methodology: sessions were applied individually or with the whole class, paper-based activities, activities with the family, continuous, specific, sequential, or ad hoc proposals. Work was carried out with the whole class, specifically seeking to strengthen rejected, neglected, or controversial students.

A copy of the programme was given to each teacher, consisting of:

- An introductory section looking at peer rejection.
- An organised calendar of the proposed activities, adapted to the Castilla y León school year calendar.
- 35 detailed files for each session (title, aims, content, description of the activity, materials, classroom layout, images/supplementary resources, etc.).
- Follow-up notebook for the intervention—specific evaluations for each activity.
- QR linked to the programme website, containing the complementary materials to download, print, or use in the IDB.
- Other useful resources: a box of programme materials (physical storybooks, lanyards for the mediators), authorisations, student success indicators, and evaluation surveys for family proposals, amongst others.

The programme was carried out together with the previously described teaching strategies, prominent amongst which are: the layout of the tables, taking into account sociometric types, encouraging students, reducing public disputes (seeking to confine these to a more private context), reinforcing empathic behaviours, developing oral communication individually and in group, and boosting the emotional expression of both teacher and student.

#### 2.3.3. Final Session (Post-Test)

This session applied the same procedure as in the initial pre-test session, working individually with each child for about eight minutes. The thoughts and impressions of the teachers were also recorded in the programme evaluation questionnaire.

#### 2.3.4. Data Analysis

Descriptive analyses were carried out to examine sociometric distribution, and the chi-squared statistic was also calculated (χ^2^) to determine whether any significant differences exist. We also analysed whether there were any differences in sociometric distribution in terms of gender and whether or not the student had specific educational support needs. We also calculated the adjusted standardised residuals (ASR), taking as a criterion significant differences in frequency if the value exceeded the range [−1.96, 1.96]. Using all of this information, we described the sample variables. We conducted *t* tests for independent samples in order to ascertain whether there were significant differences between rejected and non-rejected students and between the genders in terms of the degree of victimisation, students’ social and antisocial behaviour, and problematic social situations.

In order to validate the programme, the *t* test was used for related samples in order to determine whether there were differences between pre-test and post-test moments, both in the experimental group and in the control group. For the size effect, we calculated Cohen’s *d* (1988), taking as cut-off points (a) <0.20 as very small, (b) 0.20–0.49 as small, (c) 0.50–0.79 as moderate, and (d) >0.80 as large. Previously, we checked to see whether there were any significant differences between rejected and non-rejected students—both in the experimental group and in the control group—by using the *t* test for independent samples, calculating Hedges *g*, given the difference in the sample size of the two groups.

The IBM SPSS V.29 statistical package was used.

## 3. Results

### 3.1. Sample Scores Prior to Intervention

#### 3.1.1. Sociometric Distribution

Of the sociometric types into which the 637 students who make up the sample were distributed, we found that 8.8% of students were popular, 68.6% formed part of the average sociometric type, 10% were neglected, 2.8% were controversial students, and that 9.7% were rejected. Analysis of the sample in the experimental and in the control group reveals a similar distribution, with 9.9% of students rejected in the experimental group and 9.5% in the control group.

When analysing the general sample according to the distribution of gender, statistically significant differences emerge, χ^2^ (4, *N* = 637) = 56.086, *p* < 0.001, with a greater number of popular girls emerging—almost twice as many. Average and neglected sociometric types display a similar distribution with regards to gender. However, we see that 90.3% of the rejected students are boys. Controversial students are also predominantly male—94.4%. Girls are more popular than boys, with the latter also making up the bulk of those who are rejected and controversial.

When analysing sociometric distribution by gender—taking into account the experimental group and the control group (Table 2)—we see statistically significant differences, both in the experimental group, χ^2^ (4, *N* = 384) = 38.895, *p* < 0.001, and in the control group, χ^2^ (4, *N* = 253) = 19.701, *p* < 0.001. Moreover, we observe differences between the groups, with girls proving to be significantly more popular in the experimental group but not in the control group. Boys were found to be more rejected in the two groups. In contrast, a greater distribution of controversial boys was only significant in the experimental group.

Significant differences also emerged between those who have or do not have specific educational support needs, χ^2^ (4, *N* = 637) = 56.086, *p* < 0.001, with a significantly higher percentage of rejected students amongst those who do have special educational needs (32.6%) compared to those who do not (5%).

Prior to conducting the sociometric classroom study—and before they were aware of the distribution of each child’s profile—teachers were asked about the students who concerned them. Teachers expressed particular concern for 14.9% of the students. Nevertheless, we found that 66.3% of the students we identified as rejected had not been seen as a cause for concern by the teachers.

#### 3.1.2. Victimisation

As shown in Table 3, the level of victimisation amongst rejected students in the experimental group was differentially higher in terms of direct and indirect aggression and total victimisation compared to those who were not rejected, with moderate size effects in the three cases (*d* = 0.47, *d* = 0.42, and *d* = 0.49).

If we analyse specific behaviour for all the students, behaviours linked to hitting, pushing or kicking are those in which students are most victimised (*M* = 1.65, *SD* = 0.77). In contrast, the least common are related to manipulating classmates by controlling friendships (*M* = 1.28, *SD* = 0.57). Nevertheless, behaviours in which rejected students are most victimised, when compared to the non-rejected students, are behaviours related to “some kids in class teasing you, riling you”, t(548) = −2.99, *p* = 0.004; with a small effect size, d = 0.43; “some kids in class leaving you out of the games and not wanting to be with you”, t(549) = −2.72, *p* = 0.007, with a small effect size, d = 0.36; and “some kids in class making fun of you and laughing at you”, t(548) = −2.84, *p* = 0.005, with a small effect size, d = 0.39.

#### 3.1.3. Social and Antisocial Behaviour

Rejected students in the experimental group and in the control group present lower values for social behaviour, and higher values for antisocial behaviour when compared to non-rejected students (Table 4), with a large effect size in all cases.

#### 3.1.4. Taxonomy of Problematic Social Situations

Rejected students in the experimental group displayed higher scores in problematic social situations (Table 5) related to respecting authority and rules (*p* < 0.001, *g* = 1.16) and being disadvantaged (*p* = 0.021, *g* = 0.46), when compared to those not rejected. Something similar occurred in the control group, both with regard to respecting authority and rules (*p* < 0.001, *g* = 1.25) and in terms of being disadvantaged (*p* = 0.008, *g* = 0.67), and additionally with prosocial and empathic behaviour (*p* = 0.043, *g* = 0.52).

### 3.2. Changes in the Intervention Variables in Rejected Students after Applying the Programme

#### 3.2.1. Sociometric Distribution

After applying the programme, it was found that the experimental group had reduced the percentage of students who suffered rejection from 9.9% to 7.3%, although the same was not true of the control group, which went from 9.5% of students rejected to 10.2%. In addition, in the group that received intervention, a decrease was observed in neglected students—from 8.9% to 4.2%—while the change was not as noticeable in the control group (Table 6).

#### 3.2.2. Victimisation

Table 7 shows that, although rejected students obtained lower scores in victimisation in the post-test than in the pre-test in the experimental group, the differences were not significant. Nor were there any significant changes in the control group, even though, in this case, the post-test scores were higher than the pre-test scores.

Amongst the non-rejected children in the experimental group, a reduction was seen in the general level of victimisation, with the figure being significant, *t* = −2.32, *p* = 0.021, albeit with a very small effect size, *d* = −0.164. There was also a significant improvement in indirect aggression, *t* = −5.07, *p* = 0.000, with a small effect size, *d* = −0.38. Non-rejected students might have benefitted from the programme, both directly and indirectly, as, because the aggression levels of their classmates had fallen, the whole class felt less victimised. In contrast, and as pointed out, no improvement in victimisation was evident in the control group, with the level of both indirect and direct aggression remaining the same, and even increasing.

#### 3.2.3. Social and Antisocial Behaviour

After applying the intervention programme (Table 8), rejected students in the experimental group are seen to have significantly improved their degree of social cooperation and social interaction, albeit with a small effect size (*d* = 0.25 and *d* = 0.24, respectively), and to have reduced the externalisation of their problems (*d* = 0.13). In contrast, in the control group, significant improvement can only be seen in social cooperation, *t* = −2.36, *p* = 0.035, with a moderate effect size, *d* = −0.58. As a result, at least in this variable, the improvement in the experimental group cannot be said to be due to the intervention programme. Social cooperation is a skill which students are beginning to learn at this stage, a stage which is strongly marked as an egocentric period for children. Said skill might have improved as a result of the students’ natural process of maturing.

#### 3.2.4. Problematic Social Situations

After applying the intervention programme, rejected students in the experimental group were found to have reduced their level of affection, which means they were at a disadvantage vis à vis the peer group, with a small effect size (*d* = 0.22). No significant differences were found in the remaining factors. In contrast, rejected students in the control group showed no significant change. Indeed, the extent to which they were affected in certain problematic situations even increased, although this change did not prove to be significant (Table 9).

#### 3.2.5. Satisfaction Survey

The teachers’ evaluation survey shows that the skills which teachers feel their students can develop through the programme are self-esteem, self-knowledge, social skills, emotional regulation, reduced aggression, teamwork, assertiveness, empathy and solidarity, communication skills, conflict solving, cooperation, listening, attention, healthy peer relations, self-concept, a deeper understanding of classmates, respect for others, and peaceful coexistence, both with those they are closest to and with those they are not.

The greatest advantages that teachers found were their gaining a deeper understanding of the students, training in skills, a reliable knowledge of the relationships in the classroom and the possibility of strengthening those skills which were weak or non-existent, early intervention to prevent certain students from being rejected, an external view of the classroom that could be gained objectively and accurately using certain tools, confirming or discovering certain interactions that were unknown to them, the information received about students concerning other variables, the range of activities and their ease of use, the material resources available, and the possibility of engaging in different and innovative activities.

With regard to the difficulties and proposals for improvement, in many of their contributions, teachers highlighted a lack of time for carrying out more activities in the programme and doing so on a more regular basis, and also pointed to the time they had to devote to filling out questionnaires. They also highlighted difficulties related to age (children’s embarrassment when exposing themselves to an outsider, language barriers linked to developing maturity) or to a failure to understand the language, as well as the problem involved in combining the activities with the established curriculum they are required to complete or with finishing set schoolbooks.

Table 10 shows the responses given in the evaluation surveys. The level of satisfaction is seen to be positive, with the overall score emerging as 4.75/5.

## 4. Discussion

This study found that 9.7% of students suffered peer rejection, which is a similar percentage to that found in other studies on infant education [11]. A total of 90.3% of the children who suffered rejection are boys [16], and we found that girls were more popular than boys; 60.7% compared to 39.3% [15,16]. Social behaviour was also seen to differ between sociometric types [10], with rejected students displaying worse social behaviour, according to their teachers [10,30], and with popular children displaying the best scores in this regard.

This study found that 6.8% of students had some kind of special educational need. Of these children, 32.6% were rejected, a figure that more than doubles the rejection rate reported in the literature on non-needs students, and that concurs with other authors [14]; special needs students suffer greater rejection. It would be interesting to conduct longitudinal studies in order to determine whether this figure increases as children advance through the education system [18].

When asking the teachers in each class which students they were worried about—prior to the teachers being informed of the sociometric profiles—we found that 66.3% of those who were rejected had not been considered a cause for concern by the teachers, which highlights the subtlety involved in detecting this problem [35].

Behaviours linked to hitting, pushing or kicking are those in which students feel most victimised, with aggression being one of the main causes of rejection in childhood [25], and which concurs with the reasons students cite in their early school years for not choosing to be with certain classmates [26,27]. Nevertheless, despite these findings, we are unable to confirm that students who are most commonly victimised experience greater social rejection and isolation [44], since we found that the degree of victimisation of students rejected in the experimental group was differentially greater in terms of direct aggression, indirect aggression, and overall victimisation, compared to those who were not rejected, although we failed to find these differences in the control group.

As pointed out in the scientific literature, rejected students display poorer social behaviour. This is evidenced by the teachers’ perspective, which shows that students in the sample who do not suffer rejection display better social skills in the three variables of social cooperation, social interaction, and social independence [10,30], and that behavioural problems (externalising and internalising problems) are significantly greater amongst rejected students, such that they then exhibit fewer socially acceptable responses [19]. As they feel excluded from everyday classroom relationships, they also have to face numerous social situations which are more difficult than for students who are accepted [61], which proves that they are disadvantaged [13].

Differences were also found in terms of gender, with girls scoring much higher for social behaviour, and boys scoring much lower, with behavioural problems. All of this might further help to explain why boys are more prone to rejection (aggression, behavioural problems) and why girls are more popular, as a result of their tending to be more prosocial. It is therefore crucial to foster social skills from early childhood [51,52] in order to promote prosocial behaviour amongst all students—particularly amongst boys—and thereby reduce disruptive and aggressive behaviour.

After applying the intervention programme for socioemotional competence, the number of students rejected in the experimental group was seen to drop from 9.9% to 7.3%. In contrast, the number of students rejected in the control group—those not involved in the programme—rose slightly, from 9.5% to 10.2%. It can thus be said that putting into practice this kind of programme may prove to be very positive [55]. Indeed, it was seen that the number of positive nominations received rose slightly [46]. Implementing the intervention programme and teaching strategies was seen to promote prosocial behaviour amongst students, which has helped the children to become more popular with their classmates [50].

The anonymous satisfaction survey conducted amongst the teaching staff showed that the skills which they felt to have improved most amongst the children in the programme concur with the objectives set out herein (reduced aggression, encouraging social skills, emotional regulation, teamwork, etc.). Teachers are also very much aware that the period spanning childhood and the early teenage years are the most important in terms of learning and putting into practice these social skills [1].

We are currently at a juncture where research into social relations is sparking enormous interest [64]. Taking this into account—and the positive assessment made of the project—it is necessary to review the programme, promoting its strengths and improving on its weaknesses in order to bring us closer to achieving better prevention and reduction of peer rejection in early childhood, since this is the period in which social groups change most and in which peer rejection has not yet become chronic [13].

## 5. Conclusions

Peer rejection is a common problem in many classrooms. Yet, due to its subtle nature, it often goes unnoticed by teachers. This study describes some of the variables that may be linked to the issue, and also applies an intervention programme for socioemotional competence that boosts student development, favouring the inclusion of those who suffer rejection in the classroom. Reducing and preventing peer rejection should by no means be viewed as a secondary task. In Spain, 97% of children are already attending school by the time they reach the age associated with the second cycle of early childhood education, a figure above the OECD average (83%), such that virtually all children can be said to be in education at that stage [65]. Further to this, it is important to bear in mind that the new law on education (LOMLOE) introduces major changes to the early childhood education stage, with the aim of gradually increasing the offering of public education in the first cycle so as to thus cater to all the requests made for children aged zero to three to attend school. In fact, 45.6% of children aged from zero to three years old are enrolled in school; the highest rate ever recorded [66]. Given the high number of children from three to six years of age who are enrolled, and bearing in mind the upward trend in attending school in the first cycle of childhood, it is important to take account of the need for early intervention, since continuing with the same classmates and relationships is common in the early cycles of education between infant and primary school.

This study is also subject to certain limitations that merit highlighting. Firstly, mention should be made of COVID-19, since the early part of this project was affected by the pandemic. Some of the difficulties linked to this situation were in accessing the schools and gaining the necessary permission, the concern expressed by many families about an outsider entering the classroom, and evaluating children when taking into account the barriers to facial communication caused by having to wear face masks and by social distancing measures. A further limitation concerns the external variables that might affect the sociometric distribution found (younger children have not had the same opportunities for social relation, some children might have been overlooked by having to spend the lockdown period at home). In addition, the quality of the study would have been enriched by being able to draw on a larger sample, including being able to increase the control group. The inclusion of a placebo group would also have proved positive. Moreover, the control group differed from the experimental group in terms of the degree of victimisation. Mention should also be made of the barriers inherent to the population in which the study was carried out (the period of adaptation for first year infant pupils, the fear and embarrassment felt by certain children when faced with strangers, the lack of communication from some children, or the use of language that was hard to understand, given their age) as well as difficulties associated with language differences when dealing with foreign children. To conclude, we should perhaps also question the wisdom of offering teachers information about the sociometric types of the children in their class and of the teachers knowing—prior to the intervention programme—which students suffered rejection, as this might have led them to apply strategies or measures in a more conscious or unconscious manner to help their students, all of which might have impacted the results. The same might also be said of the control group since, although there was no intervention, teachers might have sought to alter the situation as a result of being aware of it. It would be interesting to encourage teachers to develop personal work as well as to reflect on and examine their own expectations, preconceptions, and what they offer children who behave differently—especially those who are more fidgety or inattentive—and for teachers to consider what influence all of this has on peer rejection.

For future lines of research it would be interesting to modify the intervention programme for socioemotional competence in line with the outcomes that have emerged and the criticisms put forward by the teachers, so as to improve and delve more deeply into aspects not addressed correctly or in which more specific work needs to be carried out. It is also essential to extend the time devoted to the intervention, since although improvements did emerge in certain variables, it is necessary to ascertain whether these might be enhanced even further by applying a more intensive and prolonged exposure. In addition, extending the sample to include more schools and cities may help to make the intervention more widespread, which would require making the necessary range of adaptations for the specific contexts and needs involved. Finally, it is important to follow up on the results obtained after the intervention in order to determine whether they are particular to this period or whether they remain stable over time. The programme’s design and application should be subject to constant change and improvement, since realities are ever-shifting and keeping abreast of them is vital.

Peer rejection remains a largely unknown phenomenon in society as a whole and is one which has thus far received insufficient attention in the classroom. As a result, teachers should be provided with training in order to help them pinpoint possible cases of rejection amongst those children who are prone to suffer it, and thereby prevent it from an early age. A Sustainable Development Goal (SDG 4) of the 2030 Agenda proposes that by 2030 we should “ensure inclusive and equitable quality education and promote lifelong learning opportunities for all” [67]. Preventing and reducing peer rejection may help us to take one more step towards meeting this aim.

## Figures and Tables

**Table 1:** Distribution of the sample.

	Experimental Group		Control Group	
Characteristics	*n*	%	*n*	%
Gender				
Male	195	50.8%	141	55.7%
Female	189	49.2%	112	44.3%
Course				
1st (3–4 years old)	122	31.8%	80	31.6%
2nd (4–5 years old)	121	31.5%	77	30.5%
3rd (5–6 years old)	141	36.7%	96	37.9%
School				
School 1	64	16.7%	—	—
School 2	124	32.3%	—	—
School 3	196	51.0%	—	—
School 4	—	—	52	20.6%
School 5	—	—	135	53.4%
School 6	—	—	66	26.1%
Ownership				
Public	320	83.3%	118	46.6%
Semi-private	64	16.7%	135	53.4%
SEN ^1^				
Yes	24	6.3%	19	7.5%
No	360	93.7%	234	92.5%

^1^ Note. SEN = Specific Educational Support Needs.

**Table 2:** Comparison of the distribution of sociometric types according to gender in the experimental and control groups in the pre-test stage.

Sociometric Type		Experimental		Control	
		Male (*n* = 195)	Female (*n* = 189)	Male (*n* = 141)	Female (*n* = 112)
Popular	*n* (*%*)	11 (33.3%)	22 (66.7%)	11 (47.8%)	12 (52.2%)
	ASR	−2.1	2.1	−0.8	0.8
Rejected	*n* (*%*)	33 (86.8%)	5 (13.2%)	23 (95.8%)	1 (4.2%)
	ASR	4.7	−4.7	4.2	−4.2
Average	*n* (*%*)	122 (46.4%)	141 (53.6%)	88 (50.6%)	86 (49.4%)
	ASR	−2.5	2.5	−2.5	2.5
Neglected	*n* (*%*)	14 (41.2%)	20 (58.8%)	17 (56.7%)	13 (43.3%)
	ASR	−1.2	1.2	0.1	−0.1
Controversial	*n* (*%*)	15 (93.8%)	1 (6.3%)	2 (100%)	0 (0.0%)
	ASR	3.5	−3.5	1.3	−1.3

**Table 3:** Differences in victimisation in the pre-test between rejected and non-rejected students in the experimental and control groups.

Victimisation	Experimental					Control				
	Rejected (*n* = 38)	Non-Rejected (*n* = 346)				Rejected (*n* = 24)	Non-Rejected (*n* = 229)			
	*M* (*SD*)	*M* (*SD*)	*t*	*p*	*d*	*M* (*SD*)	*M* (*SD*)	*t*	*p*	*d*
Direct aggression	8.79 (3.18)	7.16 (2.56)	−3.21	0.001	0.47	8.32 (3.28)	7.84 (2.68)	−0.660	0.510	—
Indirect aggression	4.45 (1.63)	3.93 (1.25)	−2.09	0.038	0.42	4.43 (1.82)	4.60 (1.73)	0.361	0.718	—
Victimisation	13.24 (4.36)	11.08 (3.34)	−3.20	0.001	0.49	12.75 (4.67)	12.46 (3.82)	−0.284	0.777	—

**Table 4:** Differences in social and antisocial behaviour between rejected and non-rejected students in the experimental and control groups in the pre-test stage.

Behaviour	Experimental					Control				
	Rejected (*n* = 38)	Non-Rejected (*n* = 346)				Rejected (*n* = 24)	Non-Rejected (*n* = 229)			
	*M* (*SD*)	*M* (*SD*)	*t*	*p*	*d*	*M* (*SD*)	*M* (*SD*)	*t*	*p*	*d*
Social cooperation	15.76 (5.24)	21.05 (3.46)	7.96	<0.001	1.19	12.18(5.88)	20.49 (3.94)	7.91	<0.001	1.66
Social interaction	15.54 (6.11)	19.75 (4.79)	4.36	<0.001	0.77	13.11 (5.84)	19.64 (4.48)	5.70	<0.001	1.25
Social independence	17.02 (5.03)	21.14 (3.89)	5.47	<0.001	0.92	13.83 (6.60)	20.82 (3.77)	6.86	<0.001	1.30
Externalising problems	27.49 (17.70)	10.63 (11.76)	−7.4	<0.001	1.12	33.78(16.40)	14.07 (14.78)	−5.3	<0.001	1.26
Internalising problems	9.80 (7.35)	5.86 (6.39)	−3.19	0.002	0.57	11.94 (7.77)	8.17 (7.31)	−2.01	0.045	0.50

**Table 5:** Differences in problematic situations between rejected and non-rejected students in the experimental and control groups in the pre-test stage.

Behaviour	Experimental					Control				
	Rejected (*n* = 38)	Non-Rejected (*n* = 346)				Rejected (*n* = 24)	Non-Rejected (*n* = 229)			
	*M* (*SD*)	*M* (*SD*)	*t*	*p*	*g*	*M* (*SD*)	*M* (*SD*)	*t*	*p*	*g*
Being disadvantaged	16.87(6.16)	14.46 (4.93)	−2.50	0.013	0.48	18.12 (5.56)	15.13 (4.22)	−2.70	0.008	0.67
Respect for authority and rules	8.03 (3.24)	5.19 (2.34)	−6.27	<0.001	1.16	9.32 (4.07)	5.60 (2.84)	−5.18	<0.001	1.25
Response to own success	4.81 (2.01)	4.58 (2.02)	−0.605	0.546	—	5.72 (2.95)	4.89 (2.15)	−1.51	0.133	—
Prosocial and empathic behaviour	9.53 (3.66)	8.30 (3.34)	−1.95	0.052	—	11.12 (3.22)	9.28 (3.59)	−2.03	0.043	0.52

**Table 6:** Changes in the sociometric distribution of the experimental group (*n* = 384) and control group (*n* = 253) after applying the intervention.

Sociometric Type	Experimental		Control	
	Pre-Test *n* (*%*)	Post-Test *n* (*%*)	Pre-Test *n* (*%*)	Post-Test *n* (*%*)
Popular	33 (8.6%)	18 (4.7%)	23 (9.1%)	20 (8.2%)
Rejected	38 (9.9%)	28 (7.3%)	24 (9.5%)	25 (10.2%)
Average	263 (68.5%)	306 (79.9%)	174 (68.8%)	167(68.2%)
Neglected	34 (8.9%)	16 (4.2%)	30 (11.9%)	28 (11.4%)
Controversial	16 (4.2%)	15 (3.9%)	2 (0.8%)	5 (2.0%)

**Table 7:** Differences in victimisation between the pre-test and post-test stage in rejected students in the experimental and control groups.

Victimisation	Experimental					Control				
	Pre-Test (*n* = 38)	Post-Test (*n* = 38)				Pre-Test (*n* = 24)	Post-Test (*n* = 24)			
	*M* (*SD*)	*M* (*SD*)	*t*	*p*	*d*	*M* (*SD*)	*M* (*SD*)	*t*	*p*	*d*
Direct aggression	8.52 (3.14)	7.66 (2.38)	1.13	0.270	—	8.79 (3.24)	9.14 (3.25)	−0.47	0.646	—
Indirect aggression	4.54 (1.71)	4.69 (1.78)	−0.34	0.733	—	4.64 (1.87)	5.07 (2.34)	−0.55	0.590	—
Victimisation	13.04 (4.51)	12.35 (3.49)	0.63	0.536	—	13.43 (4.60)	14.21 (5.20)	−0.62	0.547	—

**Table 8:** Differences in social and antisocial behaviour between the pre-test and post-test stage in rejected students in the experimental and control groups.

Behaviour	Experimental					Control				
	Pre-Test (*n* = 38)	Post-Test (*n* = 38)				Pre-Test (*n* = 24)	Post-Test (*n* = 24)			
	*M* (*SD*)	*M* (*SD*)	*t*	*p*	*d*	*M* (*SD*)	*M* (*SD*)	*t*	*p*	*d*
Social cooperation	15.55 (5.34)	16.78 (4.55)	−2.56	0.016	0.25	11.86 (5.42)	15.00 (5.32)	−2.36	0.035	0.58
Social interaction	15.38 (6.12)	16.73 (5.16)	−2.22	0.036	0.24	12.80 (5.29)	14.53 (5.18)	−2.04	0.060	—
Social independence	17.16 (5.10)	17.81 (4.28)	−1.28	0.211	—	13.33 (6.64)	15.46 (4.94)	−2.12	0.052	—
Externalising problems	28.58 (17.71)	26.35 (16.33)	2.12	0.043	0.13	35.47 (15.91)	28.27 (17.42)	1.60	0.131	—
Internalising problems	9.89 (7.46)	10.54 (6.11)	−0.872	0.391	—	12.29 (7.61)	13.35 (7.82)	−0.488	0.634	—

**Table 9:** Differences in problematic situations at the pre-test and post-test stage in rejected students in the experimental and control groups.

Behaviour	Experimental					Control				
	Pre-Test (*n* = 38)	Post-Test (*n* = 38)				Pre-Test (*n* = 24)	Post-Test (*n* = 24)			
	*M* (*SD*)	*M* (*SD*)	*t*	*p*	*d*	*M* (*SD*)	*M* (*SD*)	*t*	*p*	*d*
Being disadvantaged	18.07 (5.64)	15.69 (5.10)	4.18	<0.001	0.44	18.21 (5.97)	19.00 (6.48)	−0.477	0.641	—
Respect for authority and the rules	8.37 (3.30)	7.28 (2.63)	1.30	0.206	—	9.56 (3.81)	8.18 (3.76)	1.44	0.161	—
Response to own success	4.93 (2.02)	5.47 (2.13)	−1.33	0.193	—	5.81 (3.12)	6.87 (2.52)	−0.982	0.342	—
Prosocial and empathic behaviour	9.83 (3.72)	9.48 (3.85)	0.593	0.558	—	11.26 (3.41)	11.46 (4.48)	−0.152	0.881	—

**Table 10:** Teaching survey to evaluate the overall level of satisfaction with the project.

	*M*	*SD*
Knowing the sociometric distribution of my class was interesting for me.	3.90	0.301
2. The information provided to me about my students was useful.	3.81	0.402
3. I have learnt new things about peer rejection.	3.43	0.507
4. The co-operation and affability of the person who came to collect the information.	4.00	0.000
5. Their handling of the students was very good.	4.00	0.000
6. The activities are useful for working on the social skills required to prevent and reduce peer rejection.	3.48	0.680
7. I would use the activities book in future.	3.62	0.669
8. The QR and the photocopiable material help me to carry out the activities.	3.71	0.463
9. I am glad I was involved in this project.	3.76	0.436
10. This study can help to improve relations in the classroom.	3.76	0.436
11. This study has brought me closer to my students.	3.57	0.676
12. The programme has lived up to my expectations.	3.62	0.669
13. I would take part in the programme again.	3.57	0.676
14. The amount of time I had to devote to providing information was appropriate.	2.76	0.944
15. My students were happy to take part.	3.67	0.483
16. Working on peer relations at an early age is important and should be included in the infant school curriculum.	3.81	0.402
17. The activities are attractive and motivating.	3.48	0.512

Note. Range of scores [1–4].

## Data Availability

The data collected pertain to underage students and are ethically restricted.
